# Single‐cell transcriptomics reveal circulating skin‐homing CLA^+^ CTSW^+^ cytotoxic CD4^+^ T cells contribute to relapse of psoriasis

**DOI:** 10.1002/ctm2.70518

**Published:** 2025-11-17

**Authors:** Hsien‐Yi Chiu, Ka Kei Chan, Jing‐Rong Wang, Hung‐Ting Liao, Hsien‐Neng Huang, Tai‐Ming Ko

**Affiliations:** ^1^ Department of Dermatology National Taiwan University Hospital Hsin‐Chu Branch Hsinchu Taiwan; ^2^ Department of Medical Research National Taiwan University Hospital Hsin‐Chu Branch Hsinchu Taiwan; ^3^ Department of Dermatology National Taiwan University Hospital Taipei Taiwan; ^4^ Department of Dermatology College of Medicine, National Taiwan University Taipei Taiwan; ^5^ Department of Biological Science and Technology College of Engineering Bioscience, National Yang Ming Chiao Tung University Hsinchu Taiwan; ^6^ Institute of Bioinformatics and Systems Biology, College of Engineering Bioscience, National Yang Ming Chiao Tung University Hsinchu Taiwan; ^7^ Institute of Biomedical Sciences, Academia Sinica Taipei Taiwan; ^8^ Department of Pathology National Taiwan University Hospital Hsin‐Chu Branch Hsinchu Taiwan; ^9^ Department of Pathology and Graduate Institute of Pathology College of Medicine, National Taiwan University Taipei Taiwan; ^10^ Center for Intelligent Drug Systems and Smart Bio‐devices (IDS2B), National Yang Ming Chiao Tung University Hsinchu Taiwan; ^11^ School of Pharmacy, College of Pharmacy, Drug Development and Value Creation Research Center, Kaohsiung Medical University Kaohsiung Taiwan

1

Dear Editor,

Our single‐cell RNA sequencing (scRNA‐seq) profiling delineates the immune landscape that drives the relapse of psoriasis from remission following biological treatment. Psoriasis, an immune‐mediated disease, exhibits episodes of quiescence and flares of disease. Although biologic treatments have enabled remission of psoriasis, relapses following treatment cessation remain common, unpredictable and poorly understood.^1^

To investigate the role of circulating immune cells in relapse of psoriasis, we used scRNA‐seq to profile CD45^+^ peripheral blood mononuclear cells (PBMC) from patients with psoriasis vulgaris who experienced early or late relapse after achieving remission and subsequent withdrawal from biologics (Figure 1A, Figure S1, Table S1 and Supplementary Methods). We identified and annotated six major immune cell populations in PBMCs (Figure 1B–D; Figures S2 and S3, Table S2). Differentially expressed gene(s) (DEG) analysis of skin‐homing (CD4^+^ CLA (*SELPLG*)^+^) T cells from early relapsers displayed higher expressions of transcripts linked to proinflammatory responses and T cell activation, while those from late relapsers showed increased transcripts known to restrain inflammatory responses (Figure 1E–G, Figure S4 and Tables S3 and S4). Leucocyte adhesion, response to stimuli, Th17 differentiation, Th17 immune responses, T cell activation and cytokine production pathways were enriched in early relapsers (Figure 1H,I).

**FIGURE 1:**
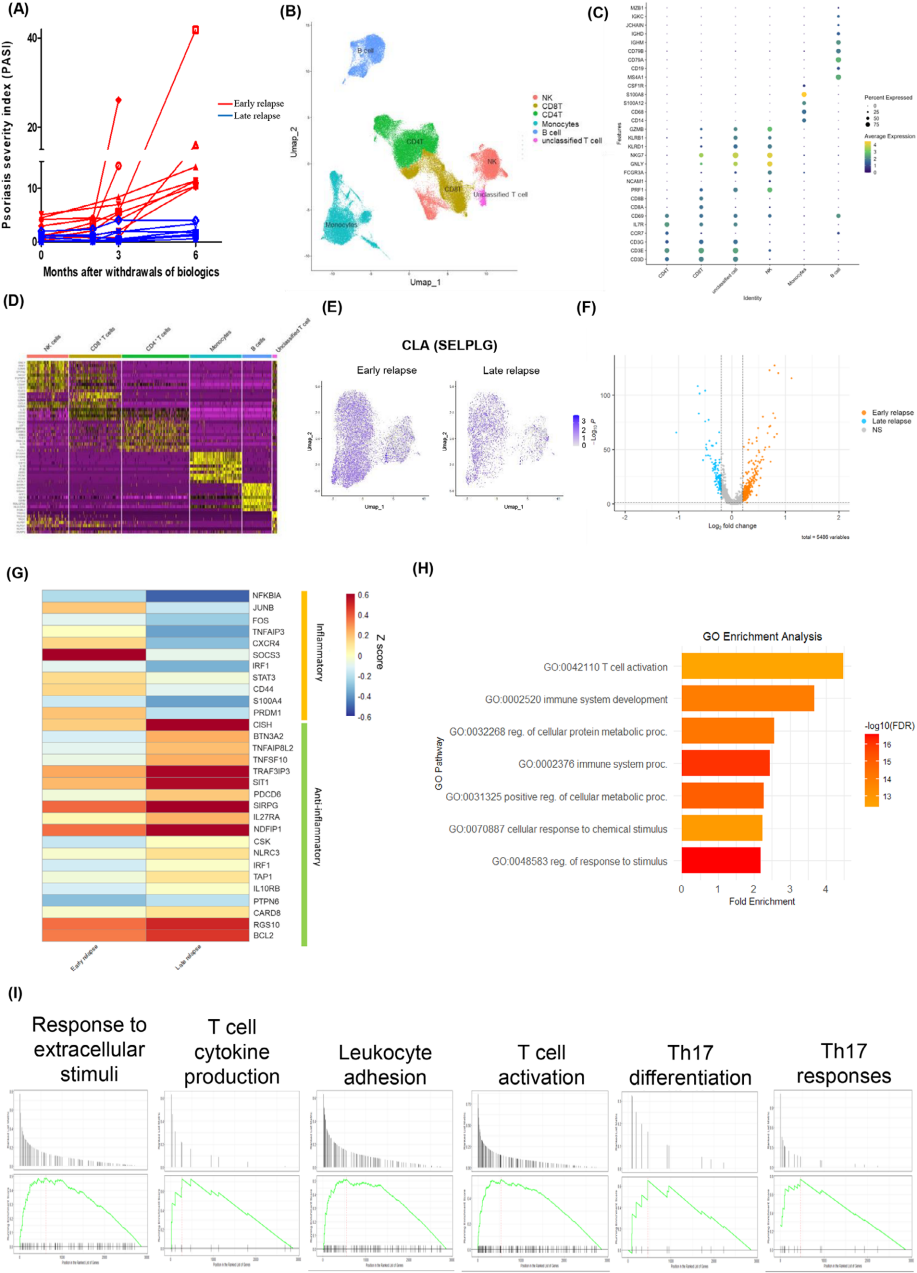
Single‐cell transcriptomic analysis reveals differential transcriptional features in skin‐homing CD4^+^ T cells between patients with psoriasis who experienced early relapse and late relapse. (A) Disease severity of psoriasis (measured by the psoriasis area and severity index, PASI) following withdrawal from biological treatment. Each line represents one patient. (B) UMAP visualization and subset annotations of scRNA‐Seq data for 78,310 PBMCs isolated at withdrawal from biologic treatment from patients who subsequently experienced early relapse (*n *= 9) or late relapse (*n *= 7). (C) Bubble plot showing the expression of marker genes used for annotation of the six major immune cell populations identified. The size of each dot represents the percentage of cells expressing the corresponding gene and the colour scale indicates the mean expression level. (D) Heatmap of the expression of the top differentially expressed genes between the six annotated major immune cell populations from (B). (E) UMAP plot illustrating the expression of the skin‐homing T‐cell marker CLA in the CD4^+^ T cell cluster. The CD4^+^ T cell cluster was defined based on the expression profiles of canonical markers and signature genes (as mentioned in the supplementary method) and CD4^+^ CLA^+^ T‐cell cluster was defined by the expression of CLA (> 0) in the CD4^+^ T cell cluster. The colour gradient reflects CLA expression, with darker blue indicating higher expression. Each point represents a single cell, with 7357 cells expressing the *CLA* gene. (F) Volcano plot of the differentially expressed genes in the skin‐homing CD4^+^ T cell cluster between the early relapse group and late relapse group (|log_2_ fold change (FC)| > 0.2 and *p*‐value < .05). The complete list of DEG is provided in the supplementary tables. (G) Heatmap showing the scaled expression (*Z*‐score) of inflammatory and anti‐inflammatory genes in CD4⁺ CLA⁺ T cells from the early and late relapse groups. The colour scale reflects the expression level, with red representing higher expression and blue indicating lower expression. (H) Gene ontology (GO) enrichment analysis of genes upregulated in CD4⁺ T cells from the early relapse group. The *x*‐axis indicates fold enrichment; the colour gradient represents the FDR‐adjusted *p*‐values, with red indicating more significant terms. (I) GSEA of enrichment of genes linked to Th17 differentiation, Th17 immune responses, and T cell activation, proliferation, and cytokine production pathways in CD4^+^ CLA^+^ T cells from early relapsers compared to late relapsers. CLA, cutaneous lymphocyte antigen; fc, fold change; FDR, false discovery rate; GOBP, gene ontology biological process; GSEA, gene set enrichment analysis; PASI, Psoriasis Area and Severity Index; PBMC, peripheral blood mononuclear cell; scRNA‐Seq, single‐cell RNA sequencing; UMAP, uniform manifold approximation and projection.

Given the pathogenic role of CD4⁺ T cells in psoriasis^1^ and to identify drivers of psoriasis relapse, we intersected DEG in CD4^+^ T with loci implicated in psoriasis by genome‐wide association studies,^2^ which showed Cathepsin W (*CTSW)* as the critical DEG (Figure 2A). CTSW is a cysteine protease from the papain family.^3^ The functions of most cathepsins are associated with antigen processing, lysosomal protein breakdown and enhanced inflammation.^3^ Skin‐homing T cells from early relapsers displayed a higher expression of *CTSW* (Figure 2B and Table S5) and higher proportions of CTSW^+^ cells compared to late relapsers (Figure 2C,D). Immunohistochemical staining revealed increased CTSW expression in T cells infiltrating psoriatic lesions of early relapsers (Figure 2E,F). However, CTSW expression was not associated with age, sex, baseline or post‐treatment psoriasis severity, or treatment modality (Table S6). Sensitivity analysis also demonstrated higher CTSW expression and a greater proportion of CTSW⁺ cells in early relapsers, consistent with the primary analysis (Figures S5 and S6).

**FIGURE 2:**
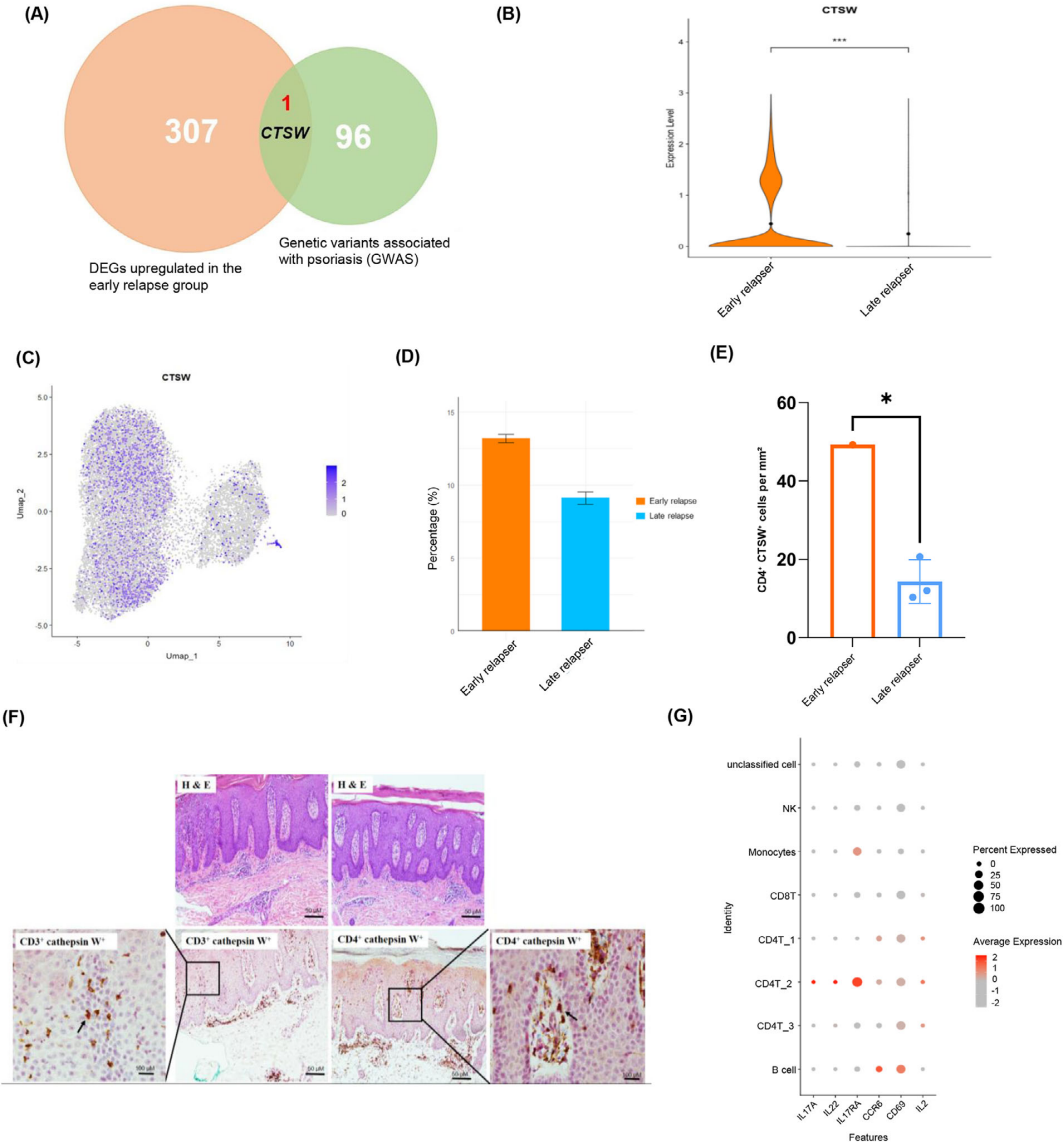
Increased abundance of circulating skin‐homing CD4^+^ CLA^+^ T cells in patients who experience early relapse. (A) Venn diagram showing the overlap between 307 upregulated DEGs in CD4^+^ T cells from the early relapse group and 96 psoriasis‐associated genetic variants; *CTSW* was identified as the uniquely common gene. The differential expression analysis of the CTSW gene between early and late relapsers yielded a raw *p*‐value of 4.16 × 10^−13^ and an adjusted *p*‐value (false discovery rate) of 1.52 × 10^−8^. (B) Violin plot illustrating higher expression of the *CTSW* gene in skin‐homing T cells from early relapse group versus late relapse group. *p < .05, **p < .01, ***p < .001 and ****p < .0001 (Wilcoxon Rank–Sum test). (C) UMAP visualization of the expression of *CTSW* in CD4^+^ T cells. The colour scale represents the expression of *CTSW*, with darker blue indicating higher expression. (D) Bar graph of the proportion of CTSW^+^ T cells in CD4^+^ T cells from patients who experienced early relapse versus patients who experienced late relapse. (E) Quantification of CD4⁺ CTSW⁺ cells per mm^2^ in psoriasis lesional skin by multiplex immunohistochemistry. *p < .05 (unpaired *t*‐test). (F) Representative images of psoriatic lesional skin stained with hematoxylin and eosin (upper panel), and multiplex immunohistochemical staining for CTSW and CD3/CD4 in a representative patient with early relapse (lower panel). Arrows in the magnified square box (right lower and left lower panels) indicate representative T cells that co‐stained for CTSW (red) and CD3/CD4 (brown). H & E, hematoxylin and eosin stain. (G) Dot plot comparing the expression of Th17‐related genes across selected cell clusters in the peripheral blood. The CD4⁺ Th17 cluster and CD4⁺ CTSW⁺ cluster were defined by the expression of Th17 signature genes and CTSW, respectively. CD4T_1 cluster: CD4⁺ T cells; CD4T_2 cluster: CD4^+^ Th17 cluster; CD4T_3 cluster: CD4^+^ CTSW^+^ cluster. CTSW, cathepsin W; DEG, differentially expressed gene; GWAS, genome‐wide association study; Th17, T helper 17 cells; UMAP, uniform manifold approximation and projection.

The low expression of Th17‐associated transcripts in the CD4⁺ CTSW⁺ T subset suggests that CTSW⁺ T cells represent a distinct T cell subset rather than Th17 cells (Figure 2G). Thus, we next compared the expression profiles of the CD4^+^ CTSW^+^ T cell subset and CD4^+^ CTSW^−^ T cell subset (Figure 3A; Tables S7 and S8). CD4^+^ CTSW^+^ T cells were enriched in transcripts linked to proinflammatory responses, whereas CD4^+^ CTSW^−^ T cells were enriched in transcripts known to negatively regulate inflammatory responses (Figure 3B,C). CD4^+^ CTSW^+^ T cells exhibited significant enrichment of pathways related to chemotaxis, leukocyte adhesion, cellular response to stimuli, cytotoxic T cells, and immune memory compared to CD4^+^ CTSW^−^ T cells (Figure 3D). Notably, CD4^+^ CTSW^+^ T cells express cytotoxic‐related genes, although at lower levels than CD8⁺ T cells (Figure 3E) and exhibited mixed expression of transcripts linked to central memory T cells (T_CM_) and effector memory T cells (T_EM_) (Figure 3F).

**FIGURE 3:**
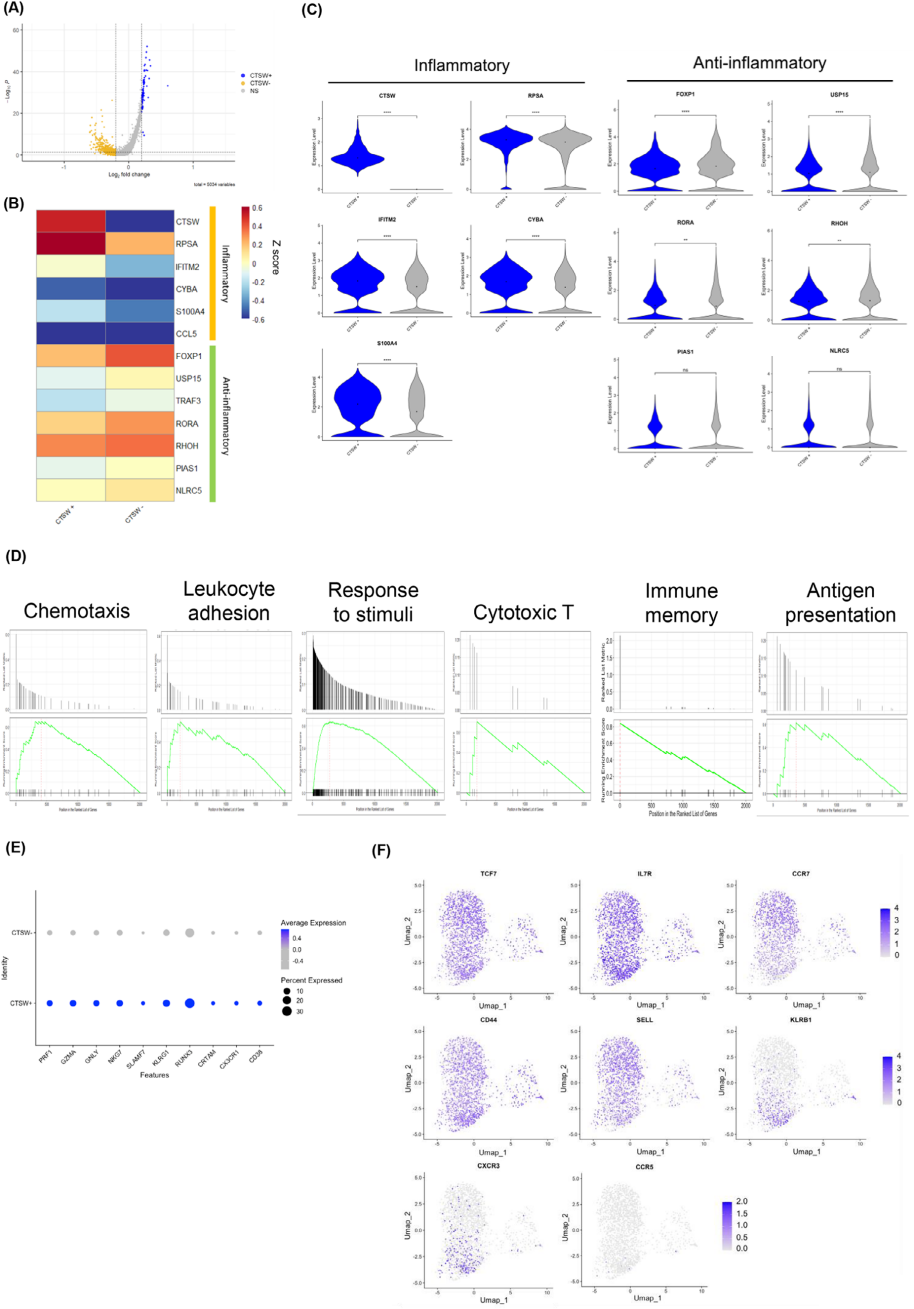
Characterization of CD4^+^ CTSW^+^ T cells. (A) Volcano plot displaying genes that were differentially upregulated in CD4^+^ CTSW^+^ T cells versus CD4^+^ CTSW^−^ T cells (|log_2_ fold change (FC)| > 0.2 and *p*‐value < 0.05). The complete list of DEG is provided in the supplementary tables. (B) Heatmap showing the scaled expression (*Z*‐score) of inflammatory genes in CD4⁺ CTSW⁺ and CD4⁺ CTSW^−^T cells. The colour scale reflects the expression level, with red representing higher expression and blue indicating lower expression. (C) Violin plots of normalized expression of example transcripts linked to inflammation and anti‐inflammatory activity between CD4^+^ CTSW^+^ T cells and CD4^+^ CTSW^−^ T cells. *p < .05, **p < .01, ***p < .001 and ****p < .0001 (Wilcoxon Rank–Sum test). (D) GSEA of enrichment of genes linked to chemotaxis, leucocyte adhesion, cellular response to stimuli, cytotoxic T cells, and immune memory pathways in CD4^+^ CTSW^+^ T cells compared to CD4^+^ CTSW^−^ T cells. (E) Bubble plot showing the mean expression (colour scale) and percentage of expressing cells (size scale) for the genes previously linked to CD4^+^ T cells with cytotoxic functions (Th‐CTXs) in CD4^+^ CTSW^+^ T cells versus CD4^+^ CTSW^−^ T cells. (F) UMAP 2D plot of the expression of genes previously linked to memory T cells in CD4^+^ CTSW^+^ T cells. The colour scale represents the expression of the indicated genes, with darker blue indicating higher expression. CTSW, cathepsin W; DEG, differentially expressed gene; fc, fold change; Th‐CTXs, CD4 T cells with cytotoxic functions; GSEA, gene set enrichment analysis; UMAP, uniform manifold approximation and projection.

Cell–cell communication analysis showed that PBMCs from early relapsers showed enrichment of communication pathways linked to inflammation and Th17 responses, whereas PBMCs from late relapsers showed enrichment of pathways associated with anti‐inflammatory responses (Figure 4A and Table S9). CD4^+^ CTSW^+^ T cells isolated from early relapsers demonstrated stronger interaction strengths with CD4^+^ Th17 cells and T_CM_ compared to late relapsers (Figure 4B). Notably, MIF signalling was enriched in interactions between CD4^+^ CTSW^+^ T cells and Th17 cells in early relapsers but absent in late relapsers (Figure 4C,D). MIF induces the production and secretion of IL‐17A and promotes the differentiation of Th17 cells,^4^, ^5^ which are major contributors to inflammation in psoriasis.^1^ In late relapsers, SIRPG/CD47 signalling was the predominant pathway enriched in the interactions between CD4^+^ CTSW^+^ T cells and Th17 cells, whereas interactions via SIRPG/CD47 signalling were absent in early relapsers (Figure 4C). Higher expression of *SIRPG* transcripts was also observed in CD4^+^ CTSW^+^ T cells from late relapsers versus early relapsers (Figure 4E). Both SIRPG and CD47 have been suggested to be negative regulators of cytotoxic T cell responses.^6^, ^7^

**FIGURE 4:**
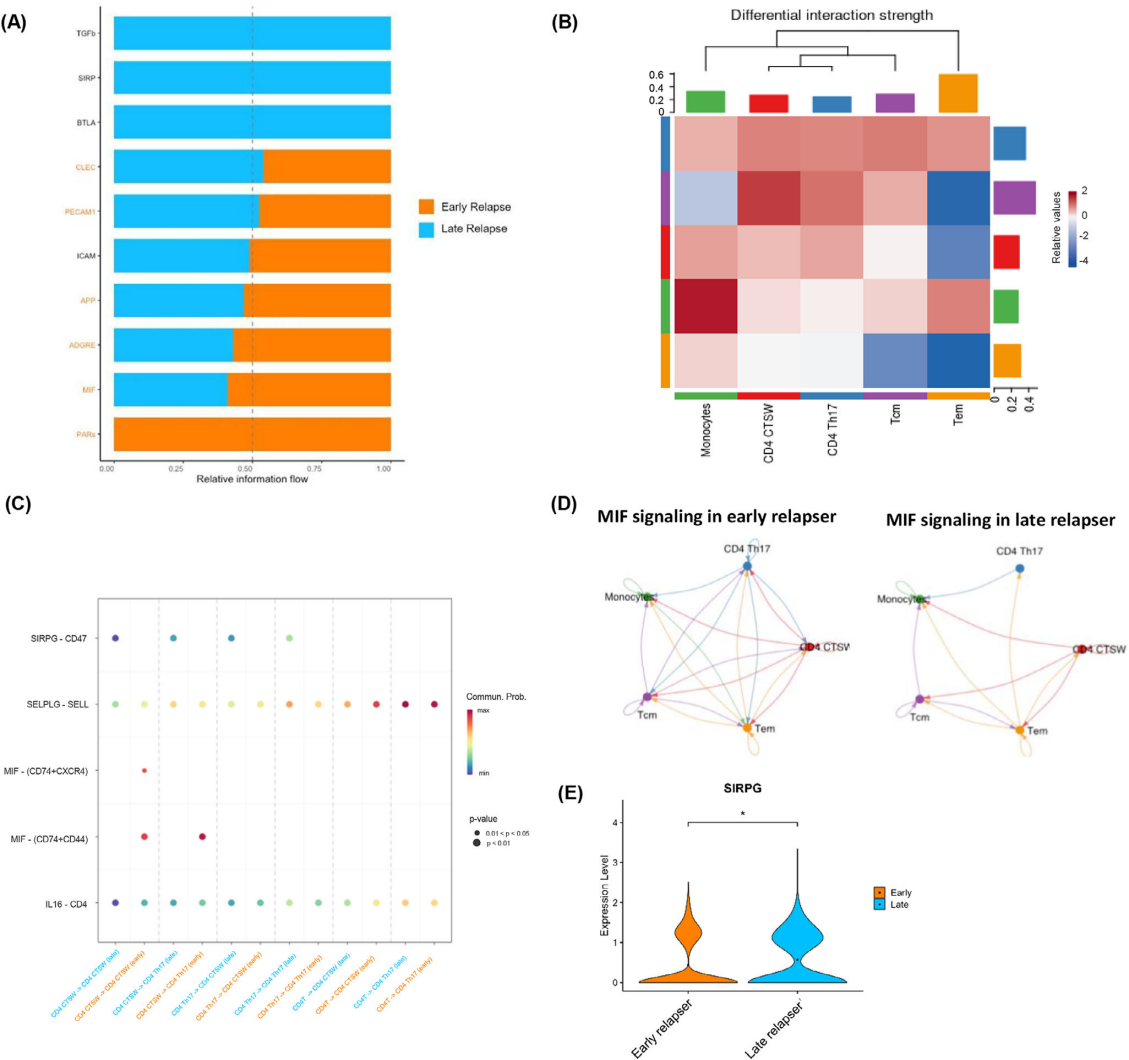
Differences in intercellular communication between immune cells in the early relapse and late relapse group. (A) Relative information flow of signalling pathways within PBMCs in the early relapse and late relapse groups. Bar plots summarize all communication probabilities in the information network of PBMCs and compare the differences in overall information flow between the early relapse group and late relapse group. Signalling pathways enriched in the early and late groups are shown in orange and blue, respectively. Please refer to Supplementary Table 6 for the abbreviations used, as well as the ligands, receptors, and features involved in each signalling pathway identified by the CellChat analysis. (B) Heatmap of the differential interaction strengths between indicated cell subsets for the early relapse group compared to the late relapse group. The colour scale reflects the relative signalling strength of a signalling pathway (early relapse vs. late relapse). The top coloured bar plot represents the sum of the column of values displayed in the heatmap. (C) Dot plot of significant ligand‐receptor interactions between the CD4^+^ CTSW^+^ T cell cluster and Th17 cell cluster in the early relapse versus late relapse group. Key ligand‐receptor interactions, including SIRPG‐CD47, SELPLG‐SELL, MIF‐ (CD74^+^ CXCR4), and MIF‐ (CD74^+^ CD44), are indicated. The size of the dots represents the significance of the interactions (*p*‐value). The *y*‐axis shows the pathways and their ligand‐receptor pairs. The colour scale reflects the communication probability (Commun. Prob.). (D) Circle plot showing the inferred intercellular communication network for MIF signalling among different cell subsets in the early relapse and late relapse groups. The size of the circle is proportional to the number of cells in each cluster and the line thickness represents the strength of signalling. (E) Violin plot illustrating higher expression of the *SIRPG* gene in CD4^+^ CTSW^+^ T cells from the late relapse group compared to the early relapse group. *p < .05, **p < .01, ***p < .001 and ****p < .0001 (Wilcoxon Ran–Sum test). CTSW, cathepsin W; Th17, T helper 17 cells; MIF, macrophage migration inhibitory factor; PBMC, peripheral blood mononuclear cell; SIRPG, signal regulatory protein‐γ; T_CM_, central memory T cells; T_EM_, effector memory T cell.

Moreover, CD4^+^ CTSW^+^ T cells from early relapsers demonstrated an increased number of communications (Figure S7A) and a stronger interaction strength with monocytes compared to late relapsers (Figure 4B). Monocytes from early relapsers showed higher expression of transcripts linked to inflammatory monocytes, inflammatory responses, T cell activation, Th17 immune response, recruitment, adhesion, and infiltration of inflammatory cells (Figure S7B,C; Figure S8 and Table S10). Conversely, monocytes from late relapsers expressed higher levels of transcripts linked to anti‐inflammatory activity and suppression of monocyte motility (Figure S7B,C; Figure S8 and Table S11). Pathway analysis revealed enrichment of responses to IL‐1, inflammatory responses, the IL‐23 pathway, response to extracellular stimuli and stress, chemotaxis, and response to IFN‐γ pathways in monocytes from early relapsers (Figure S9A,B). CellChat analysis revealed that the prostaglandin E2 (PGE2)/PTGSE3/PTGER2 (EP2) signalling pathway was significantly enriched in the interactions between CD4^+^ CTSW^+^ T cells and monocytes from early relapsers (Figure S9C). PGE2/EP2 signalling plays a critical role in IL‐23‐driven generation of pathogenic Th17 cells and development of psoriasis.^8^ Monocytes from early relapsers expressed significantly higher levels of *PTGER2* transcripts compared to late relapsers (Figure S9D). Notably, the SIRPG/CD47 signalling pathway was preferentially enriched between CD4^+^ CTSW^+^ T cells and monocytes in late relapsers, but not in early relapsers (Figure S9C). Limitation of this study included the lack of functional assays for CTSW, stratification by drug classes, and comparisons with TNF‐α‐treated, pre‐biologic and healthy cohorts. As only psoriasis patients were included, whether CTSW marks skin‐homing cytotoxic T cells in other inflammatory skin diseases warrants further investigation.

In summary, we identified a novel circulating skin‐homing CD4⁺ T cell subset, CLA⁺ CTSW⁺ T cells, that may drive psoriasis relapse by persisting in circulation, exhibiting cytotoxicity, and engaging pathogenic immune cells via MIF and PGE2/EP2 signalling, promoting a proinflammatory, Th17‐skewed milieu (Figure S10). These results provide potential targets for psoriasis relapse prevention.

## AUTHOR CONTRIBUTIONS

Hsien‐Yi Chiu, Ka Kei Chan, Jing‐Rong Wang, Hung‐Ting Laio, Hsien‐Neng Huang, and Tai‐Ming Ko contributed to the study design. Hsien‐Yi Chiu, Ka Kei Chan, Jing‐Rong Wang, and Tai‐Ming Ko collected study data. All authors contributed to data analysis or interpretation, reviewed and critically revised the manuscript, provided final approval of the version to be published, and agree to be accountable for accuracy and integrity.

## CONFLICT OF INTEREST STATEMENT

The authors declare no conflicts of interest.

## FUNDING INFORMATION

This work was supported by grants from the National Science and Technology Council, Taiwan (NSTC‐112‐2314‐B‐002‐073‐MY3 to Hsien‐Yi Chiu; NSTC‐112‐2314‐B‐A49‐039, NSTC‐113‐2314‐B‐A49‐032‐MY3 to Tai‐Ming Ko), National Taiwan University Hospital, Hsin‐Chu branch (114‐HCH001, HR‐114‐H01, and 114‐HCH034 to Hsien‐Yi Chiu), National Health Research Institutes (NHRI‐EX113‐11140SI to Tai‐Ming Ko), and the Center for Intelligent Drug Systems and Smart Bio‐devices (IDS2B) of National Yang Ming Chiao Tung University in Taiwan. The financial supporters played no part in the study design, data collection and analysis, decision to publish, or preparation of the manuscript.

## ETHICS APPRIVAL

Ethical approval was obtained from the local Institutional Review Board of National Taiwan University Hospital Hsin‐Chu branch (108‐009‐F).

## Supporting information



Supporting Information

Supporting Information

Supporting Information

Supporting Information

Supporting Information

Supporting Information

Supporting Information

Supporting Information

Supporting Information

Supporting Information

Supporting Information

Supporting Information

Supporting Information

Supporting Information

Supporting Information

Supporting Information

Supporting Information

Supporting Information

Supporting Information

Supporting Information

Supporting Information

Supporting Information

## Data Availability

Data and the original contributions related to this article are available in the Supporting Information.
