# Supplementary material for: Single‐cell transcriptomics reveal circulating skin‐homing CLA+ CTSW+ cytotoxic CD4+ T cells contribute to relapse of psoriasis
Source: Clin Transl Med. 2025 Nov 17;15(11):e70518. doi: 10.1002/ctm2.70518 (PMC12623151; doi:10.1002/ctm2.70518)
Supplement: Supplementary file 3 — Supporting Information [file CTM2-15-e70518-s021.pdf]

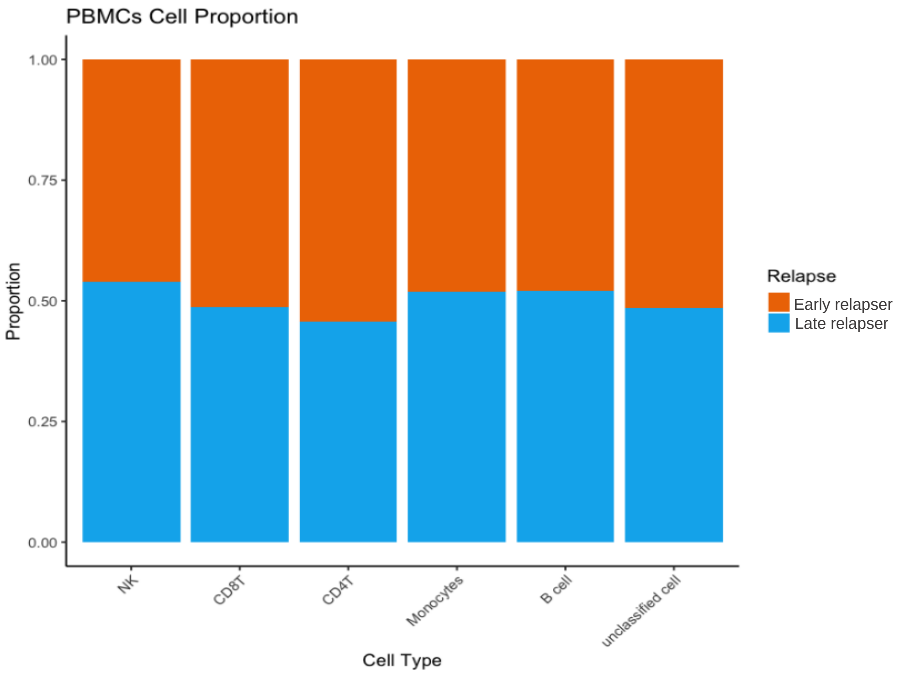

Figure S3. The proportion of the six major immune cell populations in early relapsers compared to late relapsers
