# Supplementary material for: Single‐cell transcriptomics reveal circulating skin‐homing CLA+ CTSW+ cytotoxic CD4+ T cells contribute to relapse of psoriasis
Source: Clin Transl Med. 2025 Nov 17;15(11):e70518. doi: 10.1002/ctm2.70518 (PMC12623151; doi:10.1002/ctm2.70518)
Supplement: Supplementary file 4 — Supporting Information [file CTM2-15-e70518-s007.pdf]

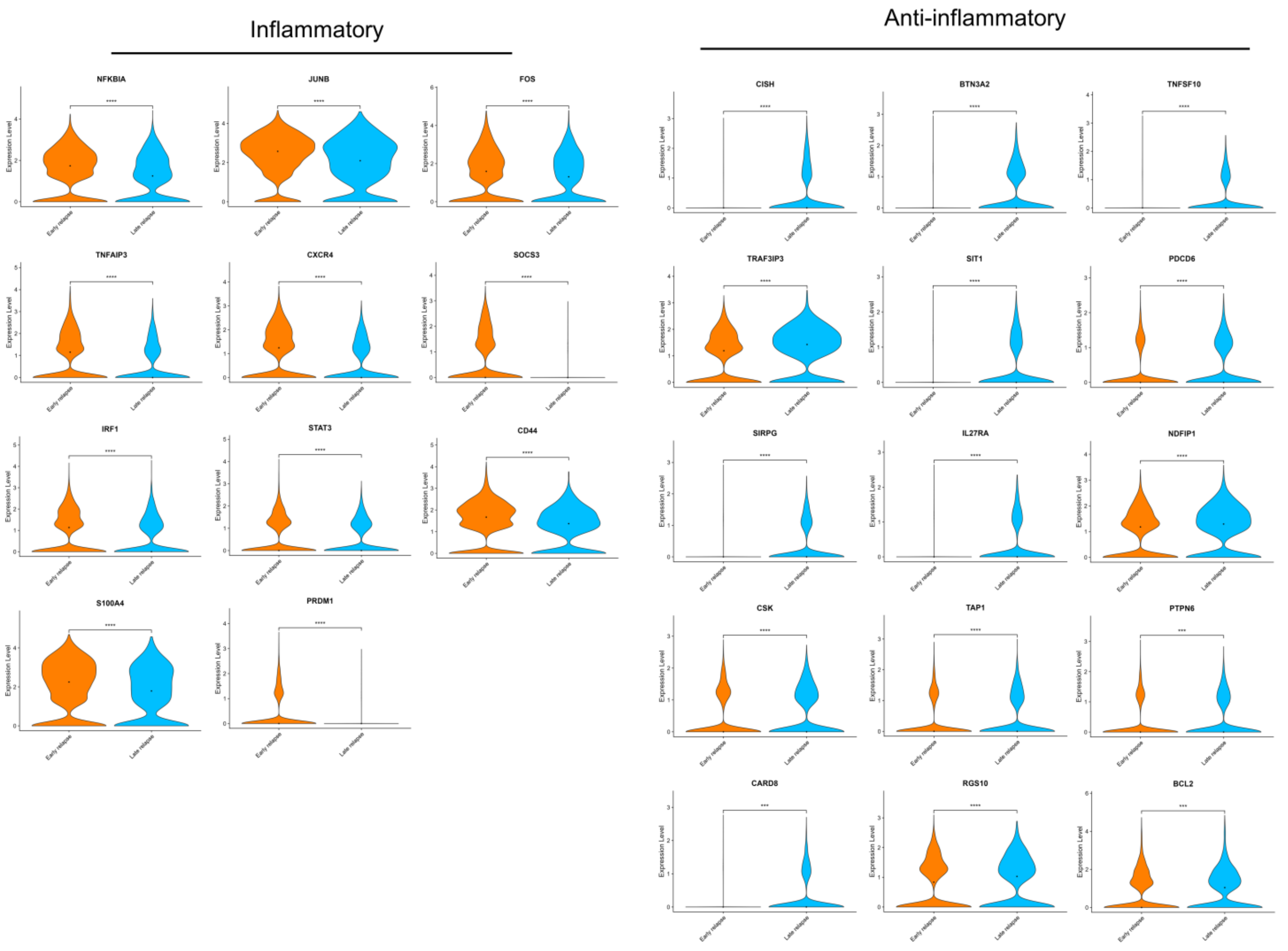

Figure S4 Differential gene expression analysis of CD4^{+} CLA^{+} T cells between early and late psoriasis relapse groups. Violin plots showing the expression levels of inflammatory and anti-inflammatory genes in CD4^{+} CLA^{+} T cells (skin-homing T cells) from early and late relapse groups. *P < 0.05, **P < 0.01, ***P < 0.001 and ****P < 0.0001 (Wilcoxon Rank Sum test).
