# Supplementary material for: Single‐cell transcriptomics reveal circulating skin‐homing CLA+ CTSW+ cytotoxic CD4+ T cells contribute to relapse of psoriasis
Source: Clin Transl Med. 2025 Nov 17;15(11):e70518. doi: 10.1002/ctm2.70518 (PMC12623151; doi:10.1002/ctm2.70518)
Supplement: Supplementary file 5 — Supporting Information [file CTM2-15-e70518-s015.pdf]

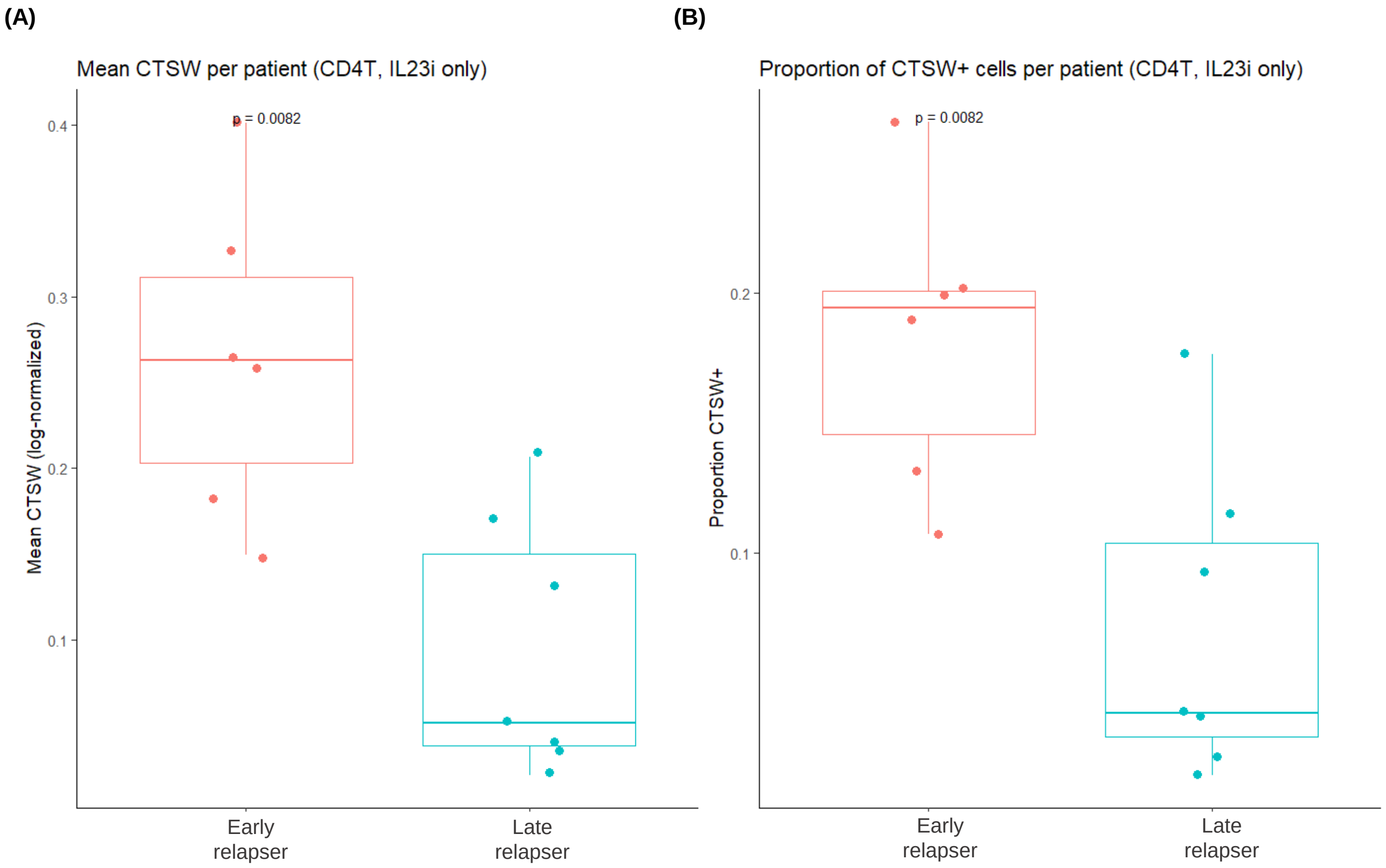

Figure S5. Results of sensitivity analyses of CTSW expression level in $CD4^+$ T cells after excluding patients who received IL-17 inhibitor treatment. CTSW, cathepsin W; IL, interleukin. Comparison between groups was analyzed using unpaired non-parametric Wilcoxon Rank Sum test.
