# Supplementary material for: Single‐cell transcriptomics reveal circulating skin‐homing CLA+ CTSW+ cytotoxic CD4+ T cells contribute to relapse of psoriasis
Source: Clin Transl Med. 2025 Nov 17;15(11):e70518. doi: 10.1002/ctm2.70518 (PMC12623151; doi:10.1002/ctm2.70518)
Supplement: Supplementary file 7 — Supporting Information [file CTM2-15-e70518-s006.pdf]

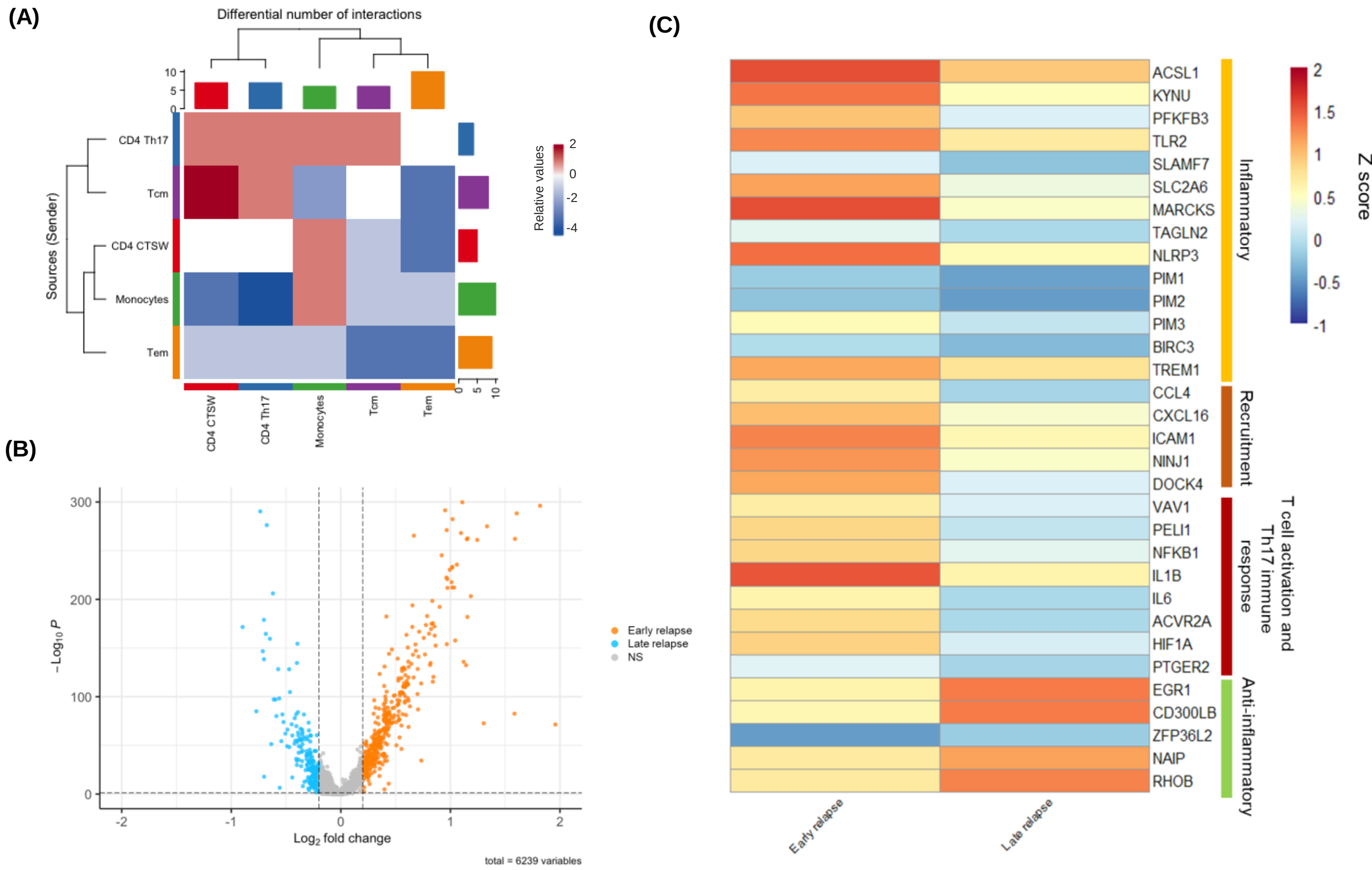

Figure S7. Characterization of the differential transcriptional features of circulating monocytes from the early relapse versus late relapse groups. (A) Heatmap showing the number of interactions of different cell subsets in PBMCs from the early relapse group compared to the late relapse group. The color scale reflects the relative differences in the number of interactions of a signaling pathway (early relapse vs. late relapse). The top colored bar plot represents the sum of column of values displayed in the heatmap. (B) Volcano plot demonstrating the differentially expressed genes between circulating monocytes from the early relapse group and late relapse group ($|\log_2$ fold change (FC)$| > 0.2$ and p-value < 0.05). The complete list of DEG is provided in the supplementary tables.(C) Heatmap of normalized expression of the indicated transcripts linked to inflammation and anti-inflammatory responses in monocytes from early relapsers compared to late relapsers. The color scale reflects the expression level, with red representing higher expression and blue indicating lower expression. CTSW, cathepsin W; fc, fold change; FDR, false discovery rate

Fig. 5
