# Supplementary material for: Single‐cell transcriptomics reveal circulating skin‐homing CLA+ CTSW+ cytotoxic CD4+ T cells contribute to relapse of psoriasis
Source: Clin Transl Med. 2025 Nov 17;15(11):e70518. doi: 10.1002/ctm2.70518 (PMC12623151; doi:10.1002/ctm2.70518)
Supplement: Supplementary file 9 — Supporting Information [file CTM2-15-e70518-s012.pdf]

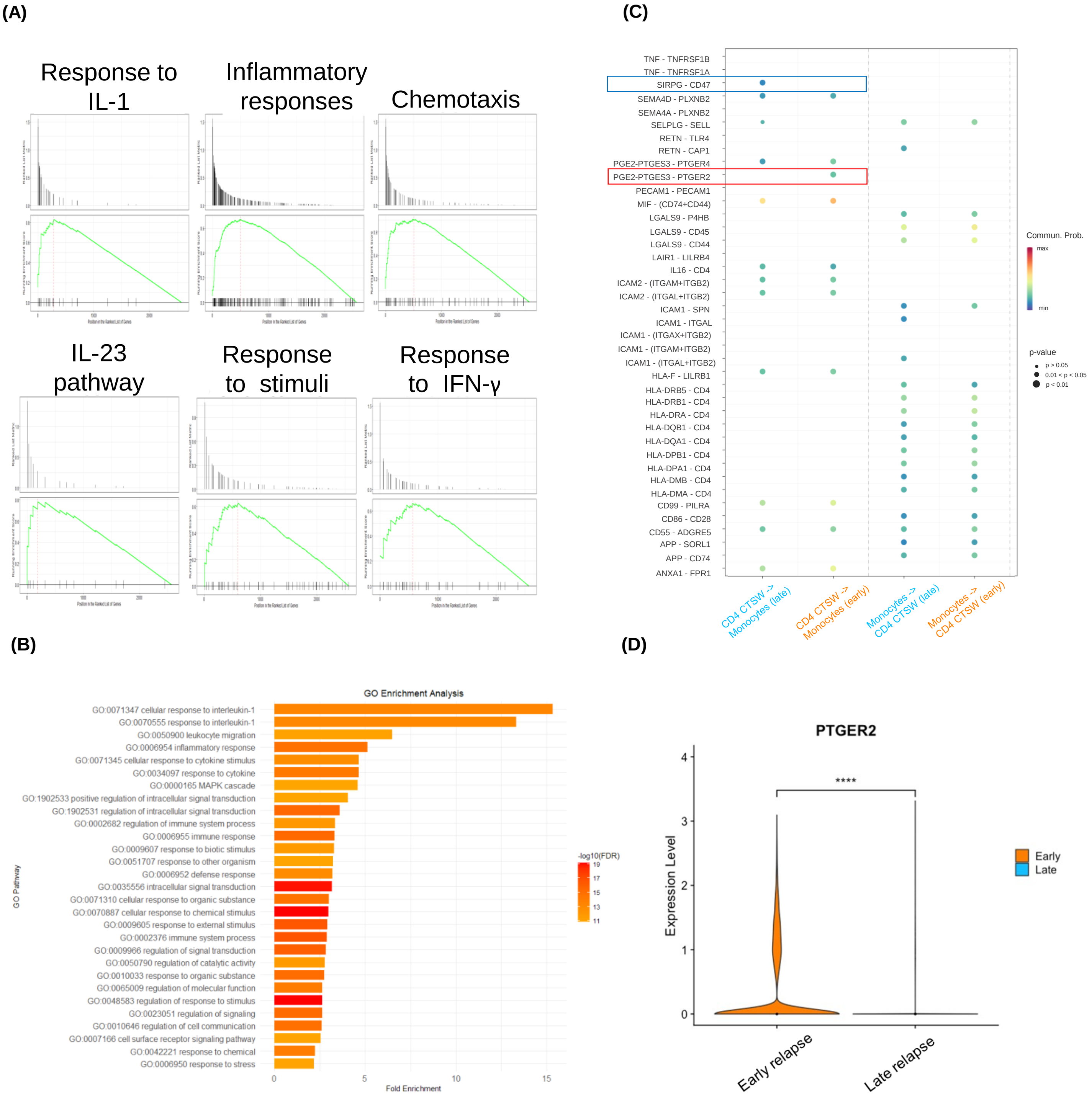

Figure S9. Pathway analysis and cell-cell communication analysis of circulating monocytes from the early relapse versus late relapse groups. (A) GSEA of enrichment of genes linked to response to IL-1, inflammatory responses, the IL-23 pathway, response to extracellular stimuli, chemotaxis, and response to IFN-γ pathways in monocytes from early relapsers compared to late relapsers. (B) Gene ontology biological process (GOBP) enrichment analysis depicting the significantly enriched biological processes in monocytes from patients who experienced early relapse. The x-axis indicates the number of genes associated with each biological process, the size of the circles reflect the fold enrichment, and the color gradient represents the FDR-adjusted p-values, with red indicating more significant terms. (C) Dot plot of significant ligand-receptor interactions between the CTSW^{+} CD4^{+} T cell cluster and monocyte cluster in the early relapse versus late relapse groups. The size of the dots represents the significance of the interactions (p-value). The y-axis shows the pathways and their ligand-receptor pairs. The color scale reflects the communication probability (Commun. Prob.). (D) Violin plot illustrating the higher expression of the PTGER2 gene in monocytes from the late relapse group compared to the early relapse group. *P < 0.05, **P < 0.01, ***P < 0.001 and ****P < 0.0001 (Wilcoxon Rank Sum test). CTSW, cathepsin W;GOBP, gene ontology biological process; GSEA, gene set enrichment analysis; IFN, interferon; IL, interleukin; PBMC, peripheral blood mononuclear cell.
