# Supplementary material for: Single‐cell transcriptomics reveal circulating skin‐homing CLA+ CTSW+ cytotoxic CD4+ T cells contribute to relapse of psoriasis
Source: Clin Transl Med. 2025 Nov 17;15(11):e70518. doi: 10.1002/ctm2.70518 (PMC12623151; doi:10.1002/ctm2.70518)
Supplement: Supplementary file 10 — Supporting Information [file CTM2-15-e70518-s014.pdf]

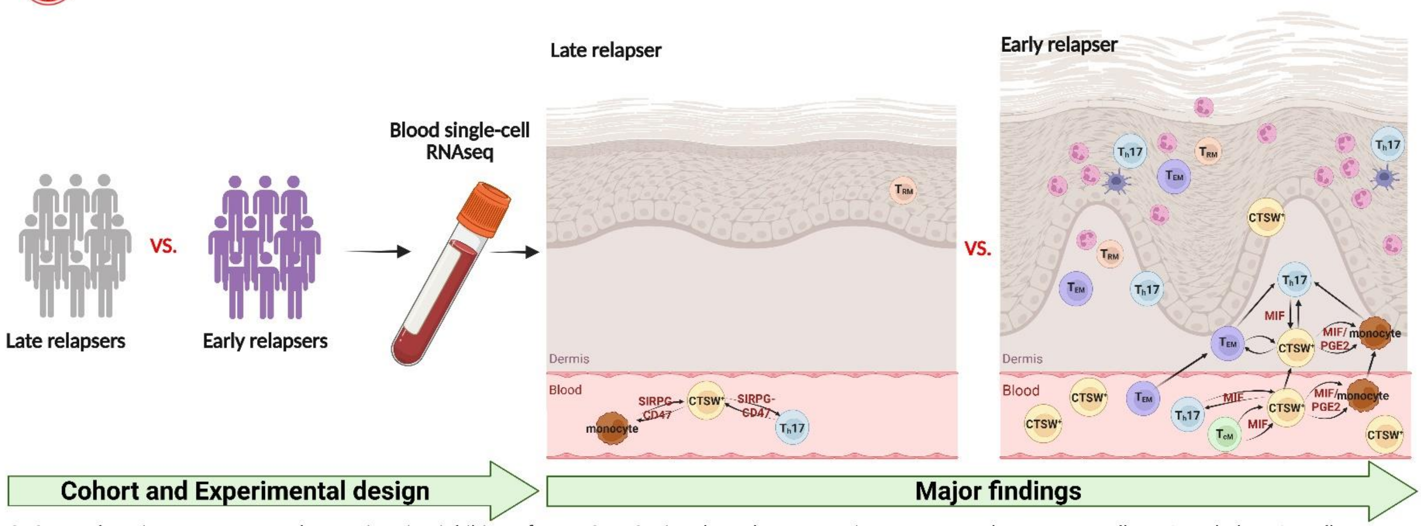


CTSW, cathepsin W; MIF, macrophage migration inhibitory factor; SIRPG, signal regulatory protein-γ; $T_{CM}$, central memory T cells; $T_h17$, T helper 17 cells; $T_{EM}$, effector memory T cell

**Figure S10**. Graphical summary. Based on comparative single-cell RNA sequencing profiling of circulating immune cells from early and late relapsers, we propose that circulating $CLA^+$ $CTSW^+$ $CD4^+$ T cells, which display cytotoxic and memory features, home to the skin upon sensing trigger stimuli and interact with monocytes, memory T cells, and Th17 cells to establish a proinflammatory, Th17-skewed milieu that drives psoriasis relapse.
