# Supplementary material for: Single‐cell transcriptomics reveal circulating skin‐homing CLA+ CTSW+ cytotoxic CD4+ T cells contribute to relapse of psoriasis
Source: Clin Transl Med. 2025 Nov 17;15(11):e70518. doi: 10.1002/ctm2.70518 (PMC12623151; doi:10.1002/ctm2.70518)
Supplement: Supplementary file 11 — Supporting Information [file CTM2-15-e70518-s013.docx]

**Supplementary Methods**

**Patient recruitment and sample collection**

A total of 16 patients with moderate-to-severe psoriasis vulgaris (defined by psoriasis area and severity index (PASI)≧10) were recruited from the dermatology department of National Taiwan University Hospital Hsin-Chu branch between September 2022 and June 2023. These patients received biologic treatments, including interleukin (IL)-23 inhibitors (guselkumab, risankizumab) or IL-17 inhibitors (brodalumab, ixekizumab), at label-indicated doses and frequencies, and achieved remission (PASI <10 and ≧50% improvement in PASI from baseline) while on biological treatments.

According to the National Health Insurance Policy of Taiwan, biological treatment is reimbursed for only two years and these patients were withdrawn from biological therapy following two years of treatment.^1, 2^ Peripheral blood mononuclear cells (PBMCs) were collected upon withdrawal from biologic treatment; all patients were in remission at that time of sampling. These patients were followed from the withdrawal of the biologic until relapse of psoriasis (PASI score ≥ 10, BSA ≥ 10%, or a loss of 50% of the improvement in PASI achieved during the preceding biological treatment).^1, 2^ The patients were classified into two groups based on the time to relapse. As previous studies reported that the average time to relapse of psoriasis was 6 months after withdrawal from biologic treatment,^1, 2^ early relapse was defined as a time to relapse < 6 months, and late relapse was defined as a time to relapse ≥ 6 months (Figure S1 and Table S1). The characteristics of study participants were presented in Supplementary Table S1. Ethical approval was obtained from the local Institutional Review Board of National Taiwan University Hospital Hsin-Chu branch (108-009-F). Peripheral blood was collected from all participants, and PBMC were isolated using Ficoll-Paque density gradient centrifugation with SepMate™ tubes, according to the manufacturer’s protocol.

**Multiplex immunohistochemistry**

Formalin-fixed, paraffin-embedded skin tissue sections (3-4 µm) from four psoriasis participants (early relapser (n = 1) and late relapsers (n = 3)) were mounted on charged slides and baked at 60 °C for 1 h. Staining was performed on an automated platform (BenchMark ULTRA, Ventana/Roche). Slides were deparaffinized, rehydrated, and subjected to heat-induced epitope retrieval with Cell Conditioning 1 (CC1; Tris-EDTA, pH ~8.5) for 64 min at 95 °C. A peroxidase block (OptiView) and protein block (casein-based) were applied according to manufacturer’s instructions.

A sequential chromogenic double-stain was used for CTSW/CD3 double stain. In round 1, T-cells were labeled with anti-CD3 (clone SP7, Sigma-Aldrich, dilution 1:150) for 32 min at 37 °C, detected with the OptiView DAB IHC Detection Kit (Ventana) to yield a brown reaction product. An on-board antibody denaturation/inactivation step (Ventana “Antibody Denaturation,” 8 min at 90–95 °C) was performed to minimize cross-reactivity in the second round. In round 2, sections were incubated with anti-CTSW (clone 1B1, Santa Cruz, dilution 1:200]) for 32 min at 37 °C and developed with the ultraView Universal Alkaline Phosphatase Red Detection Kit (Ventana) to yield a red reaction product. Slides were counterstained with hematoxylin, blued in ammonia water, dehydrated, cleared, and coverslipped. CTSW/CD4 double stain was performed by the same protocol. Anti-CD4 antibody (clone SP35, Ventana, RTU) was applied. Double-positive cells were defined as CD3-positive or CD4-positive membranes/cell outlines (brown) co-localizing with CTSW cytoplasmic/membranous red signal within the same cell profile. For each case, three non-overlapping high-power fields (HPFs, 400×; total area ~1 mm²) within regions of interest were analyzed and the amount of CD4⁺/CTSW⁺ double-positive cells per mm2 were measured.

**Single-Cell RNA sequencing (scRNA-seq)**

The 5' gene expression libraries from the single-cell samples were generated using the 10x Genomics Chromium system, where Gel Bead-In-Emulsions (GEMs) captured individual cell data. These libraries were then sequenced using the Illumina NovaSeq 6000 platform, generating high-throughput data on gene expression and library features for downstream analysis.

**ScRNA-seq alignment, preprocessing and quality control**

Raw sequencing data were processed using Cell Ranger software (v3.1.0, 10x Genomics) to convert Illumina base call (BCL) files into gene expression matrices. Reads were aligned to the human reference genome (GRCh38), resulting in gene-barcode matrices containing gene identifiers, cell barcodes, and unique molecular identifiers (UMIs). Downstream analysis was performed using the Seurat package (v4.3.0) in R. Individual datasets were merged into a single Seurat object using the merge function. Quality control (QC) procedures were applied to exclude low-quality cells and potential doublets. Cells with fewer than 200 or more than 6,000 detected genes, or with over 15% of total expression derived from mitochondrial genes, were removed. Cells with abnormally high UMI counts, were also filtered out.

Data normalization was performed using Seurat’s NormalizeData function with the default LogNormalize method and a scale factor of 10,000. To account for biases due to batch effects, we performed data integration by using Harmony batch correction with good mixing between samples.^3^ The principal components were then used for clustering via the FindNeighbors and FindClusters functions. Integrated data were generated using the IntegrateData function. Uniform Manifold Approximation and Projection (UMAP) was applied for two-dimensional visualization of the cell clusters. Initial clustering was performed using the FindClusters function in Seurat with a relatively high resolution parameter, resulting in 29 distinct clusters (Figure 1B-D and Figure S2). Cell type annotation was conducted based on the expression profiles of known marker genes using SingleR, and further validated using the CellMarker database (http://biocc.hrbmu.edu.cn/CellMarker/). Clusters corresponding to the same immune cell type were grouped together and relabeled accordingly (e.g., CD4T_1, CD4T_2). In total, six major immune cell types were identified, including CD4⁺ T cells, CD8⁺ T cells, B cells, monocytes, NK cells, and unclassified T cells (Figure 1B-D). The CD4^+^ T cell cluster was defined based on the expression profiles of canonical markers and signature genes and CD4^+^ CLA^+^ T-cell cluster was defined by the expression of CLA (>0) in the CD4^+^ T cell cluster.

**Differential expression and pathway enrichment analysis**

Differential gene expression (DEG) analysis was conducted to identify transcriptional differences within immune cell populations. Comparisons were conducted between the early and late relapse groups within CD4⁺ T cells, CD4⁺ CLA (SELPLG)⁺ T cells, and monocytes using the FindMarkers function with the Wilcoxon rank-sum test. Genes with a |log_2_ fold change (FC)| > 0.2 and *p*-value < 0.05 were considered significantly upregulated in the early relapse group, whereas genes with a log_2_ FC < -0.2 and a *p*-value < 0.05 were considered significantly upregulated in the late relapse group. In addition, we compared CD4⁺ CTSW⁺ and CD4⁺ CTSW⁻ cells to examine the differences in gene expression between these subpopulations of CD4⁺ T cells. DEG were visualized using volcano plots to illustrate the distribution of upregulated and downregulated genes. Expression levels of representative genes were further visualized using the FeaturePlot and VlnPlot functions in Seurat to highlight specific expression patterns.

Significantly upregulated genes were subjected to Gene Ontology Biological Process (GO:BP) enrichment analysis using the clusterProfiler package (v4.6.0) and ShinyGO 0.82 website (<https://bioinformatics.sdstate.edu/go/>). Pathways with a p-value < 0.05 were considered significantly enriched. Gene Set Enrichment Analysis (GSEA) was conducted using the fgsea package (v1.28.0) on pre-ranked gene lists based on log_2_ FC values obtained from the differential gene expression analysis. Gene sets from the REACTOME and Gene Ontology (GO) databases were employed for enrichment testing, and pathways with a p-value < 0.05 were deemed statistically significant.

**Cell-cell interaction network**

Cell-cell communication analysis was conducted using CellChat package version 2.1.1,^4^ which infers intercellular signaling networks based on the expression of known ligand-receptor pairs. Analyses were performed separately for the early relapse and late relapse groups of patients. First, global communication patterns among major immune cell types were assessed at the level of PBMCs. Second, a focused analysis of CD4⁺ T cell subsets was conducted, including CD4⁺ T cells, CD4⁺ CTSW⁺ T cells, CD4⁺ Th17 cells, T_CM_, T_EM_, and monocytes to investigate signaling dynamics relevant to relapse. Communication probabilities and pathway-specific information flow were inferred using standard CellChat workflows.

**Statistical method**

Differences in gene expression and cell proportions between two groups was analyzed using unpaired non-parametric Wilcoxon Rank Sum test (*P < 0.05, **P < 0.01, ***P < 0.001 and ****P < 0.0001), unless otherwise specified in the figure legend. Correlations between variables were evaluated using Kendall’s tau correlation coefficient. The statistical tests used for each panel in the figure were also described in the figure legends. All statistical analyses were conducted using The SPSS software version 19.0 (Armonk, NY, U.S.A.).

**References**

1. Chiu HY, Hui RC, Tsai TF, Chen YC, Chang Liao NF, Chen PH et al. Predictors of time to relapse following ustekinumab withdrawal in patients with psoriasis who had responded to therapy: An 8-year multicenter study. J Am Acad Dermatol 2023;88:71-8.

2. Huang YH, Hung SJ, Lee CN, Wu NL, Hui RC, Tsai TF et al. Predicting the Time to Relapse Following Withdrawal from Different Biologics in Patients with Psoriasis who Responded to Therapy: A 12-Year Multicenter Cohort Study. Am J Clin Dermatol 2024;25:997-1008.

3. Korsunsky I, Millard N, Fan J, Slowikowski K, Zhang F, Wei K et al. Fast, sensitive and accurate integration of single-cell data with Harmony. Nat Methods 2019;16:1289-96.

4. Jin S, Guerrero-Juarez CF, Zhang L, Chang I, Ramos R, Kuan CH et al. Inference and analysis of cell-cell communication using CellChat. Nat Commun 2021;12:1088.
